# Early pregnancy after bariatric surgery: a single-institute preliminary experience

**DOI:** 10.3906/sag-1909-139

**Published:** 2020-02-13

**Authors:** Emre GÜNAKAN, Hakan BULUŞ, Yusuf Aytaç TOHMA

**Affiliations:** 1 Department of Obstetrics and Gynaecology, University of Medical Sciences, Keçiören Training and Research Hospital, Ankara Turkey; 2 Department of General Surgery, University of Medical Sciences, Keçiören Training and Research Hospital, Ankara Turkey; 3 Department of Obstetrics and Gynaecology School of Medicine, Başkent University, Ankara Turkey

**Keywords:** Laparoscopic sleeve gastrectomy, obesity, pregnancy, pregnancy outcome, nutrition

## Abstract

**Background/aim:**

Pregnancy after bariatric surgery is an issue of growing importance with increasing number of women undergoing bariatric surgery. Therefore, in this study we present patients who conceived after sleeve gastrectomy and evaluate the obstetric outcomes.

**Materials and methods:**

This retrospective case-control study includes 23 women who conceived after laparoscopic sleeve gastrectomy. Patients were evaluated in two groups according to the number of months between surgery and conception (group 1: ≤12 months; group 2: >12 months).

**Results:**

The mean body mass index of patients before surgery and at the time of conception was 46.6 kg/m2 and 29.7 kg/m2, respectively. Nine patients (39.1%) had a history of infertility. There was no statistical difference between groups 1 and 2 for haemoglobin, ferritin, and 25-OH Vit-D levels or maternofoetal complication rates and pregnancy outcomes. Enteral nutrition requirements and intravenous iron replacement needs were higher in group 1, although this difference was not statistically significant.

**Conclusion:**

Pregnancy in the first years after sleeve gastrectomy seems to have similar obstetric outcomes compared to pregnancies occurring later, but it remains a controversial issue. Although the results did not have statistical significance in our study, well-designed prospective series may determine the role of enteral nutrition and intravenous iron replacement in patient management.

## 1. Introduction

The prevalence of obesity has increased over the past three decades both globally and in Turkey [1–3]. Therefore, obesity treatment has become important in recent years. The first-line treatment for obesity comprises lifestyle changes and a dietitian-controlled diet. However, some patients do not benefit from diet and may need surgical treatment. As a result, surgical treatment has become a frequently used alternative treatment choice with an increasing number of patients. Female patients constitute the majority of this patient group and more than half of them are in the reproductive period [4]. In addition, obesity is present in about 10% of pregnant women and causes serious maternal complications including gestational diabetes mellitus (GDM) and preeclampsia [5,6]. Therefore, losing weight before pregnancy is important in terms of reducing complications. 

Patients who are subjected to bariatric surgery lose weight dramatically (up to 30%) in the first year [7]. This period may be physiologically catabolic because of lower food intake or less absorption of nutrients. This catabolic process can adversely affect any possible pregnancies that may occur during this period due to maternal, fetal, or neonatal complications [8]. Therefore, patients are advised to avoid pregnancy for 12–24 months after surgery [9,10]. 

There is still no consensus in the literature on whether to expect a successful pregnancy or how long to postpone pregnancy after bariatric surgery. Therefore, in our study we aimed to compare the maternal, fetal, and neonatal outcomes of patients who conceived earlier (≤12 months) and later (>12 months) after laparoscopic sleeve gastrectomy. 

## 2. Materials and methods

This retrospective case-control study included women who conceived after laparoscopic sleeve gastrectomy surgery for morbid obesity at the Keçiören Training and Research Hospital between 2017 and 2019. All patients were informed about the risks of pregnancy and termination, and informed consent was obtained from all patients. The patients were grouped according to the number of months between surgery and conception; group 1: conceived before ≤12 months (n: 16 patients); group 2: conceived after >12 months (n: 7 patients).

### 2.1. Follow-up

Patients were followed monthly in the first trimester. Second and third trimester visits were planned as monthly, every two weeks, or weekly based on the medical status of the patient. All patients were seen by a general surgeon and obstetrician at all visits. Body mass index (BMI) values of patients before sleeve gastrectomy, at the time of conception, and at the time of delivery were recorded. All patients were subjected to laboratory tests including total blood count, liver and kidney function tests, serum electrolytes in all trimesters, ferritin and 25-hydroxy vitamin D (25-OH Vit-D) measurements in second trimester, TORCH, hepatitis B and C markers, and anti-HIV screening in first trimester. The normal ranges for hemoglobin (Hb), ferritin, and 25-OH Vit-D were 12.2–16.2 g/dL, 10–291 ng/mL, and 25–80 ng/mL, respectively. None of the patients had a prior history of diabetes. An oral glucose test (75-g OGTT) was planned for all patients in the second trimester due to their medical status. The 11–14 weeks combined test was performed for all patients for aneuploidy screening and one patient was subjected to amniocentesis.

### 2.2. Medication 

Iron supplementation (oral or intravenous) was given in all trimesters. Intravenous supplementation was planned in the case of gastric intolerance or severe anaemia. Folic acid was added to the treatment in the first trimester; vitamin D and multivitamin supplements were added in the second and third trimesters. Calcium and magnesium were replaced in case of deficiency. One patient with a history of preeclampsia and poor obstetric outcome was given low-molecular-weight heparin and acetylsalicylic acid from the beginning of the first trimester. Patients with ongoing weight loss in any of the trimesters or without weight gain at the end of the second trimester received enteral nutrition. Patients who experienced preeclampsia were treated with alpha methyldopa (alpha 2 agonist) and patients with GDM were treated with diet and insulin in case of uncontrolled blood glucose levels.

### 2.3. Bariatric surgery procedure

In all patients, surgery was completed laparoscopically. During operations, 38-Fr bougie was used. First stapler was fired beyond 3 cm of pillory and 70% of the stomach was excised by large curvature resulting in a tube-like shape.

### 2.4. Mode of delivery

Caesarean section was planned only for patients with a prior uterine surgery. Uterus and fascia were closed with an absorbable 1.0 polyglactin suture continuously, and skin with an absorbable 3.0 polyglactin suture. Vaginal delivery was the preferred approach for other patients. In episiotomy repair, vagina was closed with an absorbable 1.0 polyglactin suture continuously and perinea with an absorbable 2.0 polyglactin suture.

### 2.5. Statistical analyses

Data were analysed using SPSS 15.0 for Windows (SPSS Inc., Chicago, IL, USA). Categorical variables were compared using the chi-square test or Fisher’s exact test, as appropriate. The Wilcoxon signed ranks test was used to compare Hb levels of the first and third trimesters. Mann–Whitney U test was used to compare the birth weights of patients who conceived during and following the first year after surgery. The level of statistical significance was set at P < 0.05. 

## 3. Results 

A total of 23 patients were included in the study. The number of live births was 20. The number of patients in groups 1 and 2 was 16 and 7, respectively. The mean age of the study population was 32.4 ± 0.8 years. The mean BMI of patients before sleeve gastrectomy and at the time of pregnancy was 46.6 kg/m2 and 29.7 kg/m2, respectively. Patients had a mean weight gain of 6.3 ± 1.3 kg. Nine patients (39.1%) had a history of infertility. The mean time interval between sleeve gastrectomy and pregnancy was 11 months. The general characteristics of the patients are shown in Table 1.

**Table 1:** General properties of patients.

	Mean – sd	
Age (years)	32.4 ± 4.2	
Body Mass Index (kg/m2)(before sleeve gastrectomy)	46.6 ± 4.4	
Body Mass Index (kg/m2)(before pregnancy)	29.7 ± 3.8	
	n	%
Time after sleeve gastrectomy		
≤12 months	16	69.6
>12 months	7	30.4
Fertility status		
Primary infertile	3	13.0
Secondary infertile	6	26.1
Fertile	14	60.9
Pregnancy status		
Delivered	20	56.5
Miscarriage	3	13.0
Maternofoetal complications		
Gestational diabetes mellitus	2	10.0
Gestational hypertensive disorders	2	10.0
Preterm delivery	1	5.0
Intrauterine growth restriction	2	10.0

The mean level of Hb in the third trimester was statistically lower than in the first trimester (11.1 g/dL and 12.3 g/dL, respectively [P < 0.001]). This was also significant in separate evaluations of groups 1 and 2 (P = 0.002 and P = 0.04, respectively). There was no statistical significance between groups 1 and 2 in Hb, ferritin, or 25-OH Vit-D levels. Follow-up markers are summarized in Table 2. 

**Table 2:** Laboratory parameters.

	Group 1	Group 2	P value
Hb g/dL (1st trimester)	12.4 ± 1.2	12.3 ± 1.3	0.896
Hb g/dL (3rd trimester)	11.2 ± 0.9	10.9 ± 1.2	0.735
Fasting glucose level (mg/dL)	87.2 ± 13.2	88.0 ± 7.8	0.901
TSH level (mU/mL)	2.0 ± 1.0	1.7 ± 0.7	0.827
T4 level (ng/mL)	1.0 ± 0.15	0.93 ± 0.09	0.306
	n (%)	n (%)	
Level of Hb			
Low	9 (60.0)	3 (60.0)	0.704
Within normal range	6 (40.0)	2 (40.0)	
Level of ferritin			
Low	5 (33.3)	2 (40.0)	0.594
Within normal range	10 (66.7)	3 (60.0)	
Level of (25-OH vit-D)			
Low	10 (66.7)	2 (40.0)	0.296
Within normal range	5 (33.3)	3 (60.0)	
Fasting glucose level			
High	2 (13.3)	0	0.553
Within normal range	13 (86.7)	5 (100)	

Four patients required intravenous iron replacement, three of whom were in group 1. The reasons for intravenous replacement were gastric intolerance (n: 3) and severe anaemia (n: 1) with low ferritin level. All patients (n: 4) who required enteral nutrition were in group 1; neither the enteral nutrition requirement nor the intravenous iron replacement need were statistically significant. 

In analyses of patients who had live births, 7 patients (35.0%) had maternofoetal complications: 2 (10%) patients had GDM, 2 (10%) patients had hypertensive disorders (mild preeclampsia), 2 (10%) patients had IUGR, and 1 (%) patient had preterm delivery. One of the GDM patients was treated with diet; the other patient needed an insulin treatment in the third trimester. Patients with preeclampsia were subjected to alpha methyldopa treatment. In the analyses of maternofoetal complications, there was no statistical significance between groups 1 and 2 (33% vs. 40%, P = 0.59). 

Three patients who conceived at the 2nd, 15th, and 24th months after surgery had miscarriages in the first trimester. Two patients had a history of poor obstetric outcome; one of them had one live birth after three miscarriages and the current pregnancy resulted in an abortive outcome. The other patient had four prior miscarriages in the first trimester and two intrauterine foetal deaths in the second trimester due to preeclampsia. This patient was treated with an anticoagulant and acetylsalicylic acid during the entire pregnancy. She also experienced mild preeclampsia and was treated with alpha methyldopa at 36 weeks. She delivered vaginally by induction of labour at 37 weeks of gestation.

In analyses of foetal birth weights, there was no statistical significance between the groups (3063 g vs. 2883 g, P = 0.44). The small-for-gestational-age (SGA) birth rate was 10% (n: 2) of the total of patients. Nine patients (45%) were delivered by caesarean section. Indications were prior caesarean section in 8 patients and foetal distress in one. Two patients with hypertensive disorders and one patient with IUGR were subjected to induction of labour resulting in vaginal delivery. Surgical wound infection or episiotomy dehiscence did not occur in any of the patients. Outcomes of the pregnancies are summarized in Table 3.

**Table 3:** Pregnancy outcome.

	Group 1	Group 2	P value
Birth weight (g)	3063 ± 469	2883 ± 343	0.662
	n (%)	n (%)	
Maternofoetal complication*			
Absent	10 (66.7)	3 (60.0)	0.594
Present	5 (33.3)	2 (60.0)	
Mode of delivery			
Vaginal	8 (53.3)	2 (40.0)	0.604
Caesarean section	7 (46.7)	3 (60.0)	
OGTT			
Normal range	13 (86.7)	5 (100)	0.553
High	2 (13.3)	0 (0)	
Enteral nutrition			
Required	4 (26.7)	0 (0)	0.282
Not required	11 (73.3)	5 (100)	
Intravenous iron supplementation			
Required	4 (26.7)	1 (20.0)	0.634
Not required	11 (73.3)	4 (80.0)	

*Gestational diabetes mellitus, hypertensive disorders, intrauterine growth restriction, and preterm delivery.

## 4. Discussion

Due to the substantial number of obese women of reproductive age undergoing bariatric surgery, the issue of pregnancy after bariatric surgery began to be particularly discussed in the last decade [11]. Therefore, in this study we presented patients who conceived after laparoscopic sleeve gastrectomy, evaluated the obstetric outcomes, and obtained important findings. These data showed that earlier timing of pregnancy did not affect the obstetric outcome significantly. However, we think that this result is due to an insufficient number of patients in our study and we recommend a postponement of at least 12 months, because we think that interrupting the weight loss process and carrying a pregnancy in this catabolic period are not advisable. On the other hand, we suggest that early pregnancies may be acceptable for patients with a long history of infertility or low ovarian reserve in order to gain more time.

Obesity is associated with a large burden of medical problems, including increased maternofoetal complications. It is associated with gestational hypertension, preeclampsia, gestational diabetes, and increased birth weight [12]. In the case of medically resistant obesity, bariatric surgery seems to be a promising option for preconception management to decrease such complications [11]. Weight loss before pregnancy may have a preventive function. It is shown that obesity-related maternofoetal complications such as diabetes, hypertension or preeclampsia, and macrosomia rates significantly decrease after bariatric surgery [13,14]. On the other hand, the risk of delivering SGA infants also increases [13,14]. In this study, in the analyses of maternofoetal complications, there was no statistical significance between groups. 

The nutritional status of bariatric surgery patients is in a negative balance. These patients are also candidates for malnutrition because of lower intake due to the decreased volume of the stomach and rapid weight loss [15]. The weight loss period after bariatric surgery is most significant in the first year and guidelines recommend avoiding pregnancy for at least 12 (12–24) months after bariatric surgery [16,17]. On the other hand, it has been shown that there are similar pregnancy outcomes among women who conceive during and after the first year after bariatric surgery, as in our study [7,18]. There are limited data available on this issue because patients of reproductive age are counselled on contraception postoperatively. 

After sleeve gastrectomy, nutrition for patients begins with liquids and consists of very limited calories in the early postoperative period. This is the most catabolic period with significant weight loss [19]. Pregnancy closer to bariatric surgery may lead to more probable nutritional problems, which may occur with limited food and calorie intake. This weight loss process can be tolerated within limits in the early pregnancy period, but the calorie need increases later on. Enteral nutrition may be an option to maintain the present weight and calorie intake for patients who can tolerate only liquid nutrients. Patients with ongoing weight loss or without weight gain were administered enteral nutrition in our study. It should be noted that all patients in need of enteral nutrition were in the group that conceived in the first year after surgery, although this was not statistically significant. 

Deficiency of electrolytes (iron, calcium, etc.) and vitamins is frequently seen in sleeve gastrectomy patients and may be more apparent during pregnancy. Thus, patients who conceived after bariatric surgery should be tested and supplemented in pregnancy. The smaller surface area of the stomach and a short healing interval may result in intolerance for oral supplementation [20]. The major reason for intravenous iron supplementation in these patients was gastric intolerance rather than anaemia. Intravenous iron supplementation should be kept in mind as a possible option for these patients.

Another important point to mention is that a notable proportion of obese women have fertility problems. This rate was nearly 40% in our study. Fertility is an important reason for women to undergo bariatric surgery [21]. Thus, such patients will probably not decide to terminate the pregnancy in spite of the risks.

The standard surgical procedure and standard follow-up for all patients are considered as strengths of the present study. On the other hand, the low number of patients and retrospective design were limitations of the study. 

In conclusion, these findings are in agreement with those in the literature regarding pregnancy outcomes after bariatric surgery. Although it was not statistically significant in our study, enteral nutrition and intravenous iron replacementation may become important options in patient management in the upcoming years. In addition, although earlier timing of pregnancy did not affect the obstetric outcome significantly in our study, we still believe that interrupting the weight loss process and carrying a pregnancy in this catabolic period are not advisable. We recommend a postponement of at least 12 months, similarly to the guidelines, unless proven otherwise. On the other hand, early pregnancies may be acceptable for patients with a long history of infertility or low ovarian reserve in order to gain more time. Early pregnancy after bariatric surgery remains controversial and further prospective studies and long-term outcomes of larger series will be directive in the future.

## Acknowledgement

The authors declare that there are no conflicts of interest, financial or otherwise, related to the material presented herein. The study was approved by the hospital review board and informed consent was received from all patients. The document number is 43278876-929.
